# Linezolid Administration to Critically Ill Patients: Intermittent or Continuous Infusion? A Systematic Literature Search and Review

**DOI:** 10.3390/antibiotics11040436

**Published:** 2022-03-24

**Authors:** Ligia-Ancuta Hui, Constantin Bodolea, Laurian Vlase, Elisabeta Ioana Hiriscau, Adina Popa

**Affiliations:** 1 Pharmaceutical Technology and Biopharmaceutics Department, Faculty of Pharmacy, University of Medicine and Pharmacy “Iuliu Hatieganu”, 400012 Cluj-Napoca, Romania; ligiahui@yahoo.com (L.-A.H.); laurian.vlase@umfcluj.ro (L.V.); 2 Clinical Pharmacy Specialist, University Clinical Municipal Hospital, 400139 Cluj-Napoca, Romania; 3 ICU Department, Faculty of Medicine, University of Medicine and Pharmacy “Iuliu Hatieganu”, 400006 Cluj-Napoca, Romania; 4 ICU Department, University Clinical Municipal Hospital, 400139 Cluj-Napoca, Romania; ioanahiriscau@gmail.com; 5 Nursing Department, Faculty of Medicine, University of Medicine and Pharmacy “Iuliu Hatieganu”, 400089 Cluj-Napoca, Romania; 6 Clinical Pharmacy Department, Faculty of Pharmacy, University of Medicine and Pharmacy “Iuliu Hatieganu”, 400012 Cluj-Napoca, Romania; apopa@umfcluj.ro

**Keywords:** linezolid, continuous infusion, intermittent infusion, critically ill patients, pharmacokinetics, pharmacodynamics, clinical outcomes, side effects

## Abstract

A judicious antibiotic therapy is one of the challenges in the therapy of critically ill patients with sepsis and septic shock. The pathophysiological changes in these patients significantly alter the antibiotic pharmacokinetics (PK) and pharmacodynamics (PD) with important consequences in reaching the therapeutic targets or the risk of side effects. The use of linezolid, an oxazolidinone antibiotic, in intensive care is such an example. The optimization of its therapeutic effects, administration in intermittent (II) or continuous infusion (CI) is gaining increased interest. In a systematic review of the main databases, we propose a detailed analysis of the main PK/PD determinants, their relationship with the clinical therapeutic response and the occurrence of adverse effects following II or CI of linezolid to different classes of critically ill patients or in Monte Carlo simulations.

## 1. Introduction

The increasing microbial resistance to antibiotics represents a major public health problem with implications in determining community and nosocomial infections with enormous financial consequences [1]. According to the Center for Disease Control and Prevention’s 2019 report, 2.8 million antibiotic-resistant infections occur annually in the United States of America, and more than 35,000 people die as a consequence [2]. 

To prevent therapeutic failure and maintain germs susceptibility, optimizing the dose of antibiotics is of paramount importance, especially in critically ill patients.

Linezolid, an oxazolidinone antibiotic, is used to treat complicated pulmonary, intra-abdominal, skin, and soft tissue infections caused by methiciline-resistant *Staphylococcus aureus* (MRSA), and vancomycin-resistant enterococci (VRE). It has a unique mechanism of action by binding to the 50S subunit with inhibition of bacterial protein synthesis. This makes cross-resistance unlikely to happen. It is formulated for oral and intravenous (IV) administration [3,4]. Linezolid belongs to a time-dependent antibiotic class, where the area under the 24 h concentration-time curve at steady state divided by the minimum inhibitory concentration (AUC/MIC) > 80 and the percentage of time that the plasma concentrations surpass the MIC (T > MIC = 85–100%) were considered by many authors as optimal therapeutic pharmacokynetic/pharmacodynamic (PK/PD) indices. For the time-dependent antibiotic class, the initial dose and the total 24 h dose are important [5,6]. At the same time, the steady state concentrations (Css) of linezolid in the range of 2–10 mg/L were also associated with optimal clinical response, but the occurence of thrombocytopenia increased by 50% if Css was greater than 10 mg/L [7].

Blood and site of action concentration of a drug is influenced by its PK/PD properties, but these may vary with the patient pathophysiology. Critically ill patients have an acute health condition, which can become a chronically critical condition if they require the use of assisted medicine techniques for longer periods of time. While in healthy volunteers the PK and PD of administrated antibiotics are reasonably predictable, there is a large variability in critically ill patients [8,9].

The critically ill are affected by several changes at the same time and the linezolid PK/PD parameters are modified and may present variability from one patient to another. The multiorgan failure, hypoalbuminemia, capillary hyperpermeability syndrome associated with the use of resuscitation fluids and performing medical procedures like continuous renal replacement therapy (CRRT) or extracorporeal membrane oxygenation (ECMO) leads to larger distribution volume and larger drug clearance with a shorter half-life and risk of not achieving the therapeutic targets. Protein binding of linezolid is normally decreased (30%), and it gets lower if there is a state of hypoalbuminemia [10]. It was stated that the plasma concentration as bound and free drug may have a 5–7-fold difference between individuals and only 17% of critically ill patients have optimal minimal concentration (Cmin) continuously during a treatment. Severe liver failure may decrease the clearance by 50% [9]. In addition, the augmented renal clearance (ARC) over 130 mL/min/1.73 m^2^ increases linezolid clearance as linezolid has 30% renal clearance with the risk of subtherapeutic concentration [11]. It is known that the distribution in critically ill patients with renal failure is similar to healthy subjects, but in 11 patients with external cerebrospinal fluid drainage, the volume of distribution was more than twice the value in healthy subjects that lead to subtherapeutic plasma concentration [9].

Thirty minutes of intermittent infusion (II) of the usual dosage (600 mg every 12 h) cannot reach a drug blood concentration constantly over MIC for critically ill patients, especially for the obese, ARC, and with ECMO/CRRT. Searching for solutions for these patients is mandatory and continuous infusion (CI) may be one solution even if it is associated with therapeutic drug monitoring (TDM). CI brings benefits in efficacity as well and may be applied to time-dependent antibiotics like linezolid [11,12,13,14]. 

Several articles have proved that β-lactam CI or prolonged infusion (PI) result in better clinical outcomes in critically ill patients. In 2016, Roberts et al. conducted a meta-analysis, which included only randomized studies on critically ill patients with severe sepsis. The total number of patients studied was 632. They compared clinical outcomes after CI with II of β-lactam antibiotics, and concluded that CI of β-lactam antibiotics was associated with lower hospital mortality at 30 days and higher clinical cure compared to II [15].

In a systematic review of currently published literature, we aim to evaluate the therapeutic efficacy expressed by PK/PD indices and the clinical response and side effects following the administration of II or CI of linezolid to critically ill patients.

## 2. Materials and Methods

**Search strategy.** A systematic literature search was performed in PubMed, Embase, Web of Science, Scopus, and Cochrane, aiming to identify the studies describing the use of linezolid CI among critically ill patients with documented or suspected gram-positive infections. The keywords for literature selection used were linezolid AND (‘continuous infusion’ OR ‘prolonged infusion’ OR ‘extended infusion’ OR ‘prolonged administration’ OR ‘continuous administration’ OR ‘extended administration’ OR ‘infusion mode’) in the title, abstract or keywords. The primary goal was to identify PK/PD indices in II and CI of linezolid. As a secondary goal, in the eligible articles identified, we searched for related clinical outcomes and side effects following (II and CI) linezolid administration. The search included citations published up to the end of December 2021. 

**Selection of study.** To be eligible, the following inclusion criteria were set prior to the literature search: studies should include (1) infusion of the two daily doses of linezolid through CI in 12 h (2) in adult (3) critically ill patients with (4) documented or suspected gram-positive infections, and (5) studies should provide PK or/and PD data, or/and identified side effects and clinical outcomes. No filter was set on publication type or language. We excluded the animal studies, oral continuous administration (long term), and other antibiotics delivered through CI. 

There are several optimal PK/PD indices that are associated with clinical efficacy and toxicity. For linezolid, the clinical PK/PD indices for efficacy are AUC/MIC = 80–120 and T > MIC > 85%. As for the PK/PD threshold for haematological toxicity, we have AUC > 300 mg·h/L and Cmin > 7 mg/L [16,17]. In this analysis, we used mainly AUC/MIC > 80, T > MIC > 85%, and Cmin> 9–10 mg/L because most of the authors we cited reported these parameters. We will discuss Cmin, maximal concentration (Cmax) and Css as well, as some authors did not discuss other PK/PD indices.

**Figure 1:**
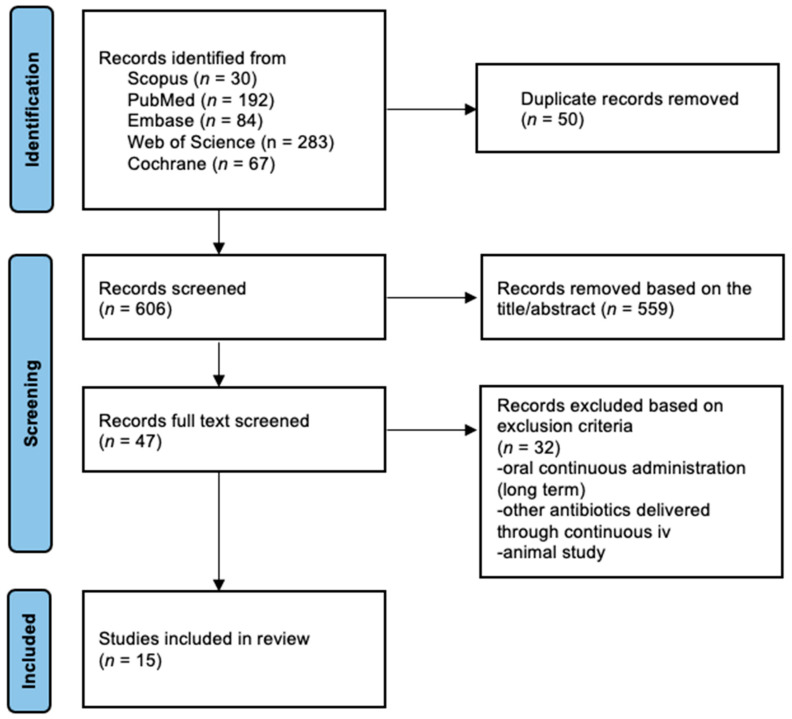
Flow chart of the review.

**Studies identified and data extraction**. The PRISMA study selection chart is shown in Figure 1. The systematic literature search was conducted by two of the authors in an independent manner up to the end of December 2021 with 656 resulting citations. After being checked for duplicates, 606 remaining citations were then checked for identifying the references relevant to the use of CI. A total of 47 articles were found to be appropriate after reading the abstracts. Finally, 15 articles discussing linezolid continuous administration via IV infusion were found to be relevant and included in the present review.

## 3. Results

The 15 eligible articles are presented in Table 1.

**Type of studies**. The studies found in the literature were in their majority prospective, open-label, randomised studies. Out of the 15 papers, 1 is a research letter, 2 are case reports, and 3 are Monte Carlo simulations (MCs). 

**Specific patient characteristic**. There are a number of patients with specific characteristics: de Pascale et al. in their publications evaluated obese patients [13,18]; Barrasa et al. included patients with ARC [11,19], Soraluce et al. patients with CRRT [14], and Kuhn et al. evaluated patients who had ECMO support [12]. The majority of patients had pneumonia due to suspected MRSA. Only Adembri et al., Tascini et al., and Alvarez-Lerma et al. used linezolid as a specific therapy (11 patients in total) [20,21,22]. Wang et al. used linezolid as a specific therapy for some of the study participants, but it was not stated for how many [6].

**Randomised research**. Eight out of the 15 studies were two-arm research with a short rate infusion of 600 mg linezolid every (q) 12 h as the control group [11,13,14,18,20,21,22,23].

**Daily dose and treatment duration.** The 15 articles that met our study criteria had over 128 patients on linezolid CI, and all of them, apart from 1 (who received 1800 mg/day [12]), received 1200 mg of linezolid during the 24 h using 2 infusions of 12 h. In 6 studies, patients received a 600 mg loading dose (79 patients) [12,13,18,19,23,26] and in 1 study they received a 300 mg loading dose (8 patients) [20] with an infusion time of 30–60 min. The rest of the authors did not use loading doses. The CI started immediately after completing the loading dose where this was used. The treatment period was stated in 10 articles, and it was at least 3 days.

**Outcomes. Pharmacokinetic aspects. Table 2** illustrates the PK/PD aspects of the studies included in the analysis. 

Css was above the 2 mg/L even for ARC and obese patients, but Cmin was in many cases below 2 mg/L. Only Barrasa et al. reported Cmin above 2 mg/L for II (patients with normal renal function). They also stated that in CI, linezolid concentration was above MIC = 2 mg/L in 94% of cases in patients without ARC, and in 70% of cases in patients with ARC [11]. They reported that the median Css was from 2.8 to 7.9 mg/L, the minimal being identified in patients with ARC, and the maximal Css was 7.9 mg/L at a normal creatinine clearance (CrCl) at 1200 mg/day dose. The median Css at 1800 mg/day was between 8.6 mg/L and 11.7 mg/L. For the II, median Cmin ranged from 0.3 to 3.3 mg/L, the first was identified in ARC patients and the maximal was reported as median Cmin in patients with CrCl from 60 to 130 mL/min/1.73 m^2^ using 1200 mg/day. When 1800 mg/day was used, a median of Cmin = 5.05 mg/L was reported in patients with CrCl between 8 and 224 mL/min/1.73 m^2^ without having the results stratified for normal renal function, medium, and severe renal disfunction. Median Cmax was 4.8–18.5 mg/L for 1200 mg/day and 16.3 mg/L at 1800 mg/day in II. Adembri et al. did not evaluate Css, only the concentration after 24 h and 72 h of infusion, and they identified a 9.1 mg/L and 10.6 mg/L, respectively, for CI. After II, they obtained only 0.65 mg/L and 2.5 mg/L at 24 h and 72 h [20].

An important parameter used to evaluate linezolid efficacy is AUC for CI, which was 146.3–189.9 mg·h/L in patients without ARC and 66 mg·h/L for ARC patients. After II, AUC varied between 110.6 and 198.4 mg·h/L in patients without ARC and 40.6 mg·h/L for ARC patients [11,13]. 

Adembri et al. found AUC/MIC ≥ 80 to be in CI 87.5%, and in II 62.5%, respectively, at MIC = 2 mg/L [20]. De Pascale et al. reported AUC/MIC ≥ 80 as being 36.3% in CI, and 18.2% in II at MIC = 2 mg/L [13].

Adembri et al., at a MIC = 1–2 mg/L, identified T > MIC > 85% as 100% for CI, and 94.3% for II [20]. De Pascale et al. had T > MIC = 100% in CI in all cases at MIC = 2–4 mg/L. In II it was only 76.1% (MIC = 2 mg/L), 44% (MIC = 4 mg/L) in the first study, and in the second one it was 82% (MIC = 2 mg/L) and 33% (MIC = 4 mg/L) [13,18]. Taubert et al. reported it as 100% in CI and 69% in II for MIC = 2–4 mg/L [27]. Wang et al. stratified data and at a CrCl < 40 mL/min/1.73 m^2^ and MIC = 4 mg/L, T > MIC > 85% was 100% in CI and 86% in II. For a CrCl = 80 mL/min/1.73 m^2^, after CI T > MIC > 85% was always achieved at 1200 mg, and after II it was achieved in only 65% of cases at MIC = 4 mg/L. For ARC patients, with II, this was not reached and for CI it was achieved in 3% of cases (MIC = 4 mg/L), 37% of cases (MIC = 3 mg/L) and 85% of cases (MIC = 2 mg/L) at 1200 mg/day and at 2400 mg/day it was 85% (MIC = 4 mg/L), and respectively 100% (MIC = 2–3 mg/L) [6]. 

Kuhn et al. conducted a study with the aim of detecting the influence of ECMO on plasma concentration for different antibiotics, including linezolid after CI of 1800 mg/day (9 ECMO patients vs. 10 non-ECMO patients). The detected median MIC for linezolid was 2 mg/L and in 35% of the ECMO patients, the target plasma concentration (6.5–12 mg/L) was not reached. In 15% of the non-ECMO patients, the MIC target was also not achieved. The median serum concentration in ECMO patients was 8.6 mg/L (5–10.5 mg/L) and in non-ECMO was 11.7 mg/L (8.3–15.4 mg/L) [12].

**Renal Data.** Barrasa et al. evaluated 26 critically ill patients with CrCl over 40 mL/min/1.73 m^2^ who received CI at a rate of 50 mg/h and obtained 94 plasma samples. Half of the patients included had ARC. The authors obtained plasma concentration in noARC group ranking from 3.3 to 18.5 mg/L and in ARC group from 0.3 to 11.9 mg/L. The plasma concentrations were above the breaking susceptibility point of 2 mg/L in 94% of samples in the noARC group and in 70% of the samples in the ARC group. In II, in the ARC group half-life, clearance, and concentration were observed to be markedly lower than in the group without ARC. In addition, the authors conducted a MCs, and the PK/PD target was higher in the CI group than the II one. The PTA (C > MIC for MIC = 2 mg/L) in the CI group was 68% at infusion rate of 50 mg/h (1200 mg/day) and 93% at 75 mg/h (1800 mg/day). For MIC = 4 mg/L, PTA was 15% at 50 mg/h and 40% at 75 mg/h, which is higher than for II. The PTA at MIC = 2 mg/L was 68% for 50 mg/h and 93% for 75 mg/h. From this point of view, the authors concluded that for ARC patients, the linezolid clearance is increased and that leads to suboptimal exposure [11].

Wang et al. used several dosage regimens in several renal function patients. Their study reported only 2% of the patients with CrCl below 40 mL/min having Css, min >10 mg/L at 600 mg q 12 h (II) vs. 56% at 1200 mg q 24 h (CI). At CrCl = 80 mL/min, they reported 0% vs. 23%, and in ARC 0% for both II and CI at the same total daily dose of 1200 mg. The study reported differences in AUC/MIC ≥ 80 and T > MIC > 85% that are listed in Table 2. For normal renal function, they found 23% cases of Css, min over 10 mg/L that could lead to higher possibility of side effects [6]. All data for CI were obtained from the MCs that was conducted after the observational study done for II, in the Wang et al. research.

The clearance of the drug seems to have the same value of 13–14 L/h independent of the regimen for normal renal function or low to moderate impairment [11,13,19,20], but in ARC, Barrasa et al. reported 21.9 L/h for CI, and 31.3 L/h for II [11].

**Outcomes. Side effects and clinical aspects.** Eight studies reported whether side effects were identified or not. Two of them described side effects in one of their patients (Table 3) [21,28]. The other six studies reported no side effects independent of the infusion regimen [11,13,14,20,22,23]. Santimaleeworagun et al. and Taubert et al. made comments on the % of patients that had Cmin > 9–10 mg/L, concentrations above which are considered toxic [24,27]. Only 3 out of the 15 studies made comments on the clinical aspects observed, which are described in Table 3 [13,22,28].

## 4. Discussion

**General pharmacokinetic parameters.** From Table 2 we can see that from the point of view of T > MIC, the CI is superior to II at MIC = 2–4 mg/L and that the differences are more obvious for MIC = 4 mg/L. In terms of AUC/MIC, the differences are less obvious. In the study by Wang et al., there are stratified data according to CrCl level. The results for AUC/MIC ≥ 80 were quite similar in each subgroup [6]. T > MIC > 85% was 100% for CI and consistently below 100% in II at MIC = 2–4 mg/L [13,18,27]. Only at MIC = 1 mg/L did II and CI have the same results [20]. Wang et al. identified no difference between CI and II at CrCl < 40 mL/min/1.73 m^2^ and reported better results for CI than II at CrCl = 80 mL/min/1.73 m^2^ [6].

For MIC = 2 mg/L, the probability of target attainment (PTA) as probability of C ≥ 2 mg/L was notably higher in CI than in II [11]. Cmin in II is below MIC = 4 mg/L in all studies with the risk of poor outcome in resistant bacteria. Cmax is over 9–10 mg/L in some cases, which is considered the risk concentration for side effects. The majority of the studies cited Cmax over 10 mg/L and Cmin below 2 mg/L, which are not appropriate for good clinical outcome and low risk of side effects, respectively.

Taubert et al. evaluated the percentage of patients with concentration between 1 and 4 mg/L (considered the effective concentration) and they concluded that 30% in CI and 0% in II had concentration constantly between 1 and 4 mg/L [27].

There are some studies that investigated 2 h, 3 h, or 4 h PI and compared it with II or CI [24,29,30,31]. Ehman et al. compared II of 30 min with 4 h PI and obtained the same results for AUC/MIC and T > MIC at 1200 mg/day, but at 2400 mg/day PI it was superior to II at MIC = 0–4 mg/L [31]. Additionally, Santimaleeworagun et al. compared II with CI and with PI. They found that the differences between CI and II are not that significant at MIC = 1 mg/L, but are more obvious at MIC = 2 mg/L. The best PTA, AUC/MIC > 80 and T > MIC > 85%, were obtained for CI, then for PI, and then for II. Regarding the risk of side effects, the risk was greater for CI, then for PI, and then for II [24]. This safety issue observed in CI may be overcome using routine TDM together with CI. The PI has not yet been studied enough to be able to formulate some conclusions.

**Renal data**. ARC can be observed in about 20% to 65% of critically ill patients. ARC is a phenomenon that could lead to PK changes like faster elimination, which could lead to sub-therapeutic concentration and consecutively poorer clinical outcomes when a standard administration regimen is used. Barrasa et al. concluded that the ARC group had significantly higher linezolid clearance, leading to lower plasma concentration and lower AUC, and that the CI regimen is a better solution than II, and for MIC above 2 mg/L, the infusion rate should be raised to 75 mg/h (1800 mg/day) [11].

The study by Wang et al. concluded that the standard dosage of 600 mg q 12 h was not optimal, and dose adjustment needs to be done based on renal function and MIC. The standard dose was adequate only for CrCl less than 40 mL/min and MIC less than 2 mg/L. T > MIC > 80% was never achieved in ARC patients neither in CI nor II at MIC = 4 mg/L at a dose of 1200 mg/day. At MIC = 2 mg/L, CI was superior to II at the same dose, reaching the target of T > MIC > 85% in ARC patients. When the dose was 2400 mg/day at MIC = 2–4 mg/L, CI reached the target, but II did not. In addition, the authors stated that for patients with ARC, the CI was essential for treatment success, but at a total dose of 2400 mg/day [6]. We consider that the Css interval closest to efficacy and with the minimum risk of side effects is between 2 and 10 mg/L. In the cited studies, Css (quantified only in the case of CI) was kept in this range. In patients with CrCl = 40–130 mL/min, Css was close to the maximum limit, and in patients with ARC close to the minimum limit, but in all cases in the range mentioned above.

Achieving the target T > MIC > 85% is influenced by renal status and MIC and the higher the kidney function and the higher the MIC, the harder it is to reach. In case of severe renal insufficiency (CrCl > 40 mL/min), this target is reached both after CI and II regardless of the MIC level. In contrast, at a CrCl = 80 mL/min, at MIC > 2 mg/L, only CI reached the target of T > MIC > 85%. In patients with ARC, II did not reach the target at all, and CI reached the target at MIC < 2 mg/L. Even though CI did not reach the target at MIC = 4 mg/L, it was still much closer to the target than II.

The drug clearance has the same value independent of the regimen for normal renal function or low to moderate renal impairment. In ARC, CI offers a lower drug clearance than II, which means higher plasma concentrations. 

**Special procedures—ECMO/CRRT.** As linezolid has a low molecular weight, low protein binding and relatively large volume of distribution, CRRT may increase its clearance and in association with the physiological changes found in critically ill patients, linezolid blood concentration may have large variations. It is known that subtherapeutic concentrations lead to failure of antimicrobial therapy and antibiotic resistance. For an 80% eradication rate, AUC/MIC should be 100 and 600 mg q 12 h in a 30-min infusion failed to achieve that [32]. Soraluce et al. evaluated patients with CRRT and II and AUC/MIC ≥ 80 was only 52 for MIC = 2 mg/L and 0 for MIC = 4 mg/L. Unfortunately, there are no data for CI in CRRT patients. In noCRRT patients, the differences are significant in favor to CI [14]. We can conclude that CI in noCRRT patients is superior to II, and this is more obvious in MIC = 4 mg/L. As there were no data provided for CI in CRRT patients, we cannot formulate any conclusion. 

In the study by Kuhn et al., the target plasma concentration was not reached more often in ECMO patients than non-ECMO ones and even though some of the patients had CRRT too, the authors concluded that their statistical analysis did not provide evidence that the low serum concentration can be attributable to CRRT [12]. From this data, we can conclude that for ECMO patients there are limited data regarding the comparison of CI with II. If CI is used in patients with ECMO and/or CRRT, routine TDM is required to avoid subtherapeutic concentrations.

**Pulmonary diffusion.** Alveolar diffusion was evaluated by Boselli et al. and De Pascale et al. as epithelial lining fluid linezolid concentration (LNZ_ELF)_ calculated as LNZ_ELF_ = LNZ_BAL_ (linezolid concentration from a bronchoalveolar lavage) x urea dilution index (plasma urea concentration/BAL urea concentration). This is important to note because it provides information on the ability of cultures negativation and therefore a clinical cure. They included in their studies 30 critically ill patients with pneumonia, and 24 of them had LNZ_ELF_ evaluation. De Pascale et al. included obese patients weighing ≤150 kg, but Boselli et al. did not report having any obese patients, enrolled or not. Boselli et al. concluded that II provides alveolar concentrations exceeding 4 mg/L only 75% of the time in comparison with CI, which provided alveolar concentrations over 4 mg/L, 100% of the time. De Pascale et al. also conducted a MCs and concluded that CI is associated with a higher ELF/plasma ratio percentage than II with ELF Css above 4 mg/L in all patients including the obese patients. Both studies reported similar Css, AUC and alveolar diffusion (97%) for the CI regimen, but De Pascale et al. stated that this similarity may not be true in morbidly obese patients [13,23]. According to these studies’ results, we may conclude that CI has a pulmonary penetrability superior to the one offered by II. This leads to a greater capacity of culture negativation obtained by BAL. The alveolar diffusion of linezolid has not been influenced by obesity, but it is not known whether morbid obesity could have a negative impact on it.

**Clinical outcomes.** Three studies reported better clinical outcomes as clinical improvement and mortality for CI [13,22,28]. The other authors did not evaluate these clinical aspects. De Pascale et al., 2015, reported a significant decrease of ICU mortality, at 9% after CI from 36.4% after II [13]. Alvarez-Lerma et al. observed that after CI, there is a negativation in pulmonary cultures and CI favors the weaning of invasive mechanical ventilation, a fact not seen after II, in their case report [22].

**Side effects.** There are few studies that evaluate linezolid side effects for CI, but some data exists for II. It was stated that patients with renal impairment and/or low serum albumin have a higher drug blood concentration and that linezolid main metabolite accumulates, which may lead to side effects, like haematological toxicity. Patients undergoing haemodialysis have a bigger chance of developing haematological and metabolic complications, which are dose dependent. It was stated that treatment longer than 11–15 days and Cmin > 8.2 mg/L increase the risk of thrombocytopenia. Fang et al. concluded that AUCday1 ≥ 163 mg·h/L, AUCday2 ≥ 207 mg·h/L, steady state AUC (AUCss) ≥ 210 mg·h/L, and Cmin, day2 ≥ 6.9 mg/L, minimal concentration at steady state (Cmin ss) ≥ 6.9 mg/L are good indicators for the toxicity risk [33].

In the studies included in our research, Tascini et al. reported one case of thrombocytopenia in II regimen and Protti et al. reported one case of severe refractory lactic acidosis without hypoxia in CI regimen (Table 3) [21,28]. Six other studies reported no side effects independent of the infusion regimen [11,13,14,20,22,23]. Taubert et al. evaluated the percentage of patients with a concentration constantly over 10 mg/L, which was considered the toxic concentration and it was identified in 16% of CI and 4% of II [27]. Santimaleeworagun et al. reported C > 9 mg/L as a toxic concentration, which was identified in 99.6% cases for CI and 14.3% for II. Their recommendation was to use PI of 4 h for MIC = 1–2 mg/L, and synergic antibiotic association for greater MICs [24]. The rest of the authors did not make any comments on side effects. 

**Monte Carlo simulations.** We identified three MCs that evaluate the CI utility. Taubert et al. obtained concentrations over MIC more often in CI than II. Wang et al. observed that for ARC patients, 600 mg two times a day administered through II does not provide efficacious drug blood concentrations, and CI of 2400 mg/day offered the best results [6,27]. The other articles that are prospective studies are in line with the MCs mentioned above, stating that II does not provide good drug blood concentration and that CI seems to provide higher and more stable drug blood concentration. Barrasa et al. (a prospective study) included ARC patients as well, and concluded that CI of 50 mg/h (1200 mg/day) provides good blood concentration for noARC situations, but not enough for ARC, and 75 mg/h (1800 mg/day) should be considered; Wang et al. (MCs) recommended 100 mg/h (2400 mg/day) [6,11,19].

As a consequence of this data, we propose that TDM should be the standard of care for most antibiotics used in the ICU and it is therefore recommended to use routine TDM for linezolid together with CI in the critically ill patients. Using this method, the sub- and over-therapeutic concentration may be avoided. This may offer better clinical outcomes and lower risk of side effects [16]. 

**Challenges encountered with CI**. When using CI, one concern is the linezolid stability at room temperature for longer periods of time, for example 12 h or 24 h. In the product specifications, the manufacturer specifies that the linezolid premix ready to use solution is valid for 24 h after unsealing [34]. Taylor et al. concluded that linezolid stability in isotonic natrium chloride 0.9% and glucose 5% at 25 °C is kept for 34 days [35]. 

Another issue is the possibility of drug-drug interaction especially through Y site catheter. Linezolid is incompatible in solution with amphotericin B, diazepam, dantrolene, daunorubicin, erythromycin lactobionate, garenoxacin, pantoprazole, thiopental, phenytoin, chlorpromazine, pentamidine, and sulfamethoxazole/trimethoprim [4,36,37]. There are several websites with free or paid access that have lists of linezolid compatibility like www.micromedex.com (accessed on 5 May 2021), www.medscape.com (accessed on 5 May 2021) or www.stabilis.org (accessed on 5 May 2021), and nursing staff can check the compatibilities. In the ICU where CI is usually used, this information can be provided by clinical pharmacists. 

Another concern is the accidental rapid infusion with the risk of overdosing, but with an intelligent and secured automated pump and well-trained nursing staff, this can be overcome. 

In addition, this brings difficulty in moving the patient and performing physical therapy and other procedures and investigations. Nevertheless, this type of infusion is used in the vast majority of critically ill patients, who have a limited degree of travel regardless and who are assisted by allied health or nursing staff and only when strictly necessary. To overcome this challenge, a movable stand can be used, or a 22 h infusion can be used. When the infusion is stopped for a longer period, a new loading dose is needed [38,39]. 

A CI is easier to handle for a nurse as she has to apply it once or twice a day and it operates automatically. The drug monitoring is easier to do as the concentration measurement is done at a steady state at any point of time and the plasma concentration must be 4- or 5-fold of the MIC [26].

Our study limitations are that we found a relatively small number of articles having research letters, case reports and research published in abstract volume papers included as well. The identified articles have a relatively reduced number of patients included and inhomogeneous records on PK/PD parameters. Another issue is that there is almost no data concerning the clinical outcome and side effects observed during linezolid CI.

## 5. Conclusions

Intermittent administration of linezolid is shown to be suboptimal in achieving PK/PD indices in critical care patients, leading to the risk of therapeutic failure and promoting bacterial resistance. However, these shortcomings can be overcome by CI of linezolid, the main beneficiaries being patients with preserved renal function, those with ARC and those with obesity. In addition, CI of linezolid is even more effective at elevated bacterial MIC levels. The efficacy of CI of linezolid remains unclear in patients with ECMO and/or CRRT. 

However, due to some variability in plasma concentrations following CI, in order to avoid the administration of subtherapeutic or toxic doses, TDM monitoring is required.

Regarding the reporting of the clinical response and the incidence of side effects during CI, the data are much poorer in comparison with II, and it is imperative that new studies be conducted to identify and quantify them.

Linezolid remains a vital antibiotic in the treatment of infections with resistant gram-positive germs and its use in a judicious manner is necessary.

## Figures and Tables

**Table 1:** Selected studies description.

Reference	Study Type	Antibiotic Group		Treat-Ment Duration	Case Definition	Authors Stated Recommendation/ Observation
		CI Group	II Group			
Santimaleeworungan et al., 2021 [24]	MCs on 10,000 subjects based on 318 pts in a PK study	MCs of various dosing regimens including CI of 1200 mg/24 h	318 pts with 600 mg q 12 h through oral or 30 min–2 h IV [25]	Up to 3 months	Critically ill patients with VRE, MRSA or other gram infections	‘Even 600 mg every 8 h and 1200 mg as a CI gave a higher target attainment of AUC/MIC and a T > MIC and the target cumulative fraction response (CFR), but those regimens gave Ctrough ≥ 9 mg/L rates of 40.7% and 99.6%. The current dosing of 1200 mg/day might be an optimal treatment regimen for VRE infections with MIC ≤ 1 mg/L for documented therapy, whereas the standard dose of 600 mg infused in 4 h every 12 h might be considered as optimal regimen for empirical treatment against VRE infection’.
Wang, et al., 2021 [6]	Prospective, observational, multi-center, open-label, two arm	MCs of 1200 mg/2400 mg q 24 h	117 pts with 600 mg q 12 h	8 days	ICU Chinese adult critically ill with pneumonia (the majority) or other infections (bloodstrem, CNS, bone and joint, skins and soft tissue). CrCl = 8.74–222.4 mL/min. Documented or empiric therapy	‘In critically ill patients, the standard dose of 600 mg q 12 h was sufficient for MIC ≤ 2 mg/L in patients without ARC. Moreover, a 2400 mg/day 24 h CI was recommanded for ARC patients’
Barrasa et al., 2020 [11]	Prospective, open-label, multi-center, two arm + MCs on 1000 subjects	26 pts with 1200 mg CI/day	17 pts with 30 min IV of 600 mg q 12 h	At least 7 days	Critically ill with CrCl ≥ 40 mL/min, but 32% with ARC (≥165 mL/min). Empiric therapy	‘This study shows that ARC significantly increases linezolid CL and leads to a high risk of suboptimal exposure when the standard dose is used. CI may be a useful strategy to increase the probability of treatment success, becoming one of the few options for patients with ARC. To ensure drug C > 2 mg/L in these pts, a higher infusion rate (75 mg/h) should be considered’.
Soraluce et al., 2020 [14]	Prospective, open-label, multi-center, two arm	11 pts with 1200 mg CI/day	40 pts with 30 min IV of 600 mg q 12 h	Not stated	Critically ill with (23 pts) or without (17 pts) CRRT	‘Our study confirmed that the standard regimen of linezolid may be insufficient to reach the PK/PD target to cover infections caused by pathogens with MIC > 2 mg/L. The administration of linezolid as CI instead of II notably increases the achievement of PK/PD target’
Bohle et al., 2020 [26]	Prospective, observational single center, single arm	25 pts with 600 mg IV loading dose + 1200 mg CI/day	ND	Not stated	ICU patients	‘For the drug of last resort, linezolid, underdosing seems to be more common than overdosage’
Kuhn et al., 2020 [12]	Prospective, comparative, observationalsingle center, single arm	19 pts with 600 mg IV loading dose + 1800 mg as CI/day	ND	Not stated	ICU patients with severe respiratory and bloodstreem infections. Empiric and documentated G+ infections	‘Our observations suggest that continuous application of linezolid can be successfully employed in ECMO patients. However, TDM is necessary and should regularly be carried out when linezolid is administered. Further studies are warranted to assess different dosing regimens for anti-infective drugs in patients on ECMO support, and these should prospectively compare CI versus II of selected antibiotics’.
Taubert et al., 2017 [27]	MCs based on a Prospective, observational, single center, single arm	Simulation of 67,000 pts based on 52 pts with different infusion regimens (30 min IV q 6 h, q 8 h, q 12 h or CI) using covariate characteristic from 134 pts (28 with ARDS)		At least 4 days	Critically ill pts with severe infections	‘CI provide best target attainment rates with regards to T > MIC, but their use should be evaluated very carefully due to a presumably elevated risk of toxicity and mutant selection in critically ill patients’.
Barrasa et al., 2017 [19]	Prospective, multi-center, single arm. Poster	22 pts wih 600 mg IV loading dose + 1200 mg as CI/day	ND	At least 4 days	Critically ill with CrCl ≥ 40 mL/min, but 32% with ARC (≥165 mL/min). Empiric therapy	‘Despite the high CrCl values of the patients, 50 mg/h linezolid CI ensures a high probability of achieving the PK/PD target if CrCl < 165 mL/min. In the presence of CrCl > 165 mL/min, a higher dose should be considered’
Protti et al., 2016 [28]	Case report	1 pt with 1200 mg CI/day	ND	5 days	Post transplant pneumonia patient	‘Linezolid-induced lactic acidosis is associated with diminished global oxygen consumption and abnormally high venous oxygen saturation.’
Alvarez-Lerma et al., 2016 [22]	Case report	1 pt with 1800 mg CI/day	1 pt with 60 min IV of 600 mg q 12 h	35 days	Septic shock secondary to community acquired MRSA pneumonia	‘In ICU patients with severe infections and increased renal clearance, linezolid should be administered at high doses and in CI with close monitorization of plasma drug levels’
De Pascale et al., 2015 [13]	Prospective randomised, controlled, two arm + MCs on 1000 situations	11 pts with 600 mg IV loading dose + 1200 mg as CI/day	11 pts with 60 min IV of 600 mg q 12 h	At least 3 days	Critically ill obese patients with VAP. Empirical therapy	‘In critically ill obese patients affected by VAP, linezolid CI may overcome the limits of standard administration, but these advantages are less evident with difficult to treat pathogens (MIC = 4 mg/L). These data support the usefulness of linezolid CI, combined with TDM, in selected critically ill population’.
De Pascale et al., 2013 [18]	Prospective, randomised, single center, two arm. Abstracts volume	7 pts with 600 mg IV loading dose + 1200 mg as CI/day	7 pts with 600 mg IV q 12 h	At least 3 days	Critically ill obese patients with nosocomial pneumonia due to suspected MRSA	‘Despite the optimal pulmonary penetration, linezolid plasmatic concentrations may be suboptimal in obese critically ill patients treated by II. CI would be able to overlap this limit, but clinical studies are needed in order to confirm these preliminary PK data’.
Boselli et al., 2012 [23]	Prospective, open-label, single center, single arm	12 pts with 1 h IV of 600 mg, followed by 1200 mg as CI/day plus a β-lactam and amikacin	ND	Not stated	Critically ill adult patients with suspected VAP, with CrCl ≥ 40 mL/min. Empiric therapy	‘1200 mg of intravenous linezolid administered CI to critically ill patients with VAP should be effective against organisms with MICs as high as 2–4 mg/L. However, further study is needed to determine not only the optimal PK/PD target when using linezolid in CI during the treatment of VAP, but also the clinical benefit of CI in comparison with II’.
Tascini et al., 2011 [21]	Research letter	2 pts with 600 mg IV q 12 h and who continued with 1200 mg as CI/day.	8 pts with 600 mg IV q 12 h	10–47 days	Endocarditis in patients with native or prosthetic valve or pacemaker with documented MRSA, MSSA, MRSE, VRE	‘The elevated levels of linezolid in CI achieved may explain the positive outcome. Linezolid may be used as rescue therapy in difficult-to-treat patients who have endocarditis’
Adembri et al., 2008 [20]	Prospective, open-lable, randomised, single center, two arm	8 pts with 30 min IV of 300 mg + 900 mg as CI in day 1, followed by 1200 mg as CI	8 pts with 30 min IV of 600 mg q 12 h	Not stated	Septic critically ill ICU adult patients with documented G+ glycopeptide non-responsive infection	‘Time that the free drug concentration was above the MIC (Tfree > MIC) of >85% was more frequent in CI than in II (*p* < 0.05). Finally, with CI it was possible to achieve AUC/MIC values of 80–120 more frequently than with II (*p* < 0.05). Further studies with a larger number of patients are necessary to demonstrate the possible clinical benefit and the safety of this administration modality’

Pt(s)—patient(s), IV—intravenous, CI—continuous infusion, II—intermittent infusion, CrCl—creatinine clearance, q—every, VAP—ventilation associated pneumonia, ND—not determined, ICU—intensive care unit, CRRT—continuous renal replacement therapy, G+—gram positive bacteria, MRSA—methicillin-resistant *Staphylococcus aureus*, MRSE—methicilline-resistant *Staphylococcus epidermidis*, MSSA—methicillin-sensitive *Staphylococcus aureus*, VRE—vancomycin resistant *Enterococcus,* TDM—therapeutic drug monitoring, CL—clearance, ARC—augmented renal clearance, ECMO—extracorporeal membrane oxygenation, MCs—Monte Carlo simulation, ARDS—acute respiratory distress syndrome, C—concentration, PK—pharmacokinetic.

**Table 2:** Pharmacokinetic/pharmacodynamic results for every type of infusion.

Reference	Type of Infusion	Pharmacokinetic/Pharmacodynamic Parameters						
		Css (mg/L)	Cmax (mg/L)	Cmin (mg/L)	PK/PD Indices (Targets)			
					AUC/MIC ≥ 80	MIC (mg/L)	T > MIC ≥ 85 (%)	MIC (mg/L)
Santimaleeworagun et al., 2021 [24]	CI 1200 mg q 24 h				100 99.9	1 2	100	1–2
	II 600 mg q 12 h				97.1 66.1	1 2	91.2 72.2	1 2
Wang et al., 2021 [6]	II CrCl = 8–224		16.3	5.05	-	-	-	-
	II CrCl < 40	-	-	-	100 66 20	2 3 4	100 98 86	2 3 4
	CI CrCl < 40	-	-	-	100 66 22	2 3 4	100	2–4
	II CrCl = 80	-	-	-	74 23 0	2 3 4	94 75 65	2 3 4
	CI CrCl = 80 1200 mg/24 h	-	-	-	72 23 0	2 3 4	100	2–4
	II ARC 1200 mg/24 h	-	-	-	0	2–4	0	2–4
	CI ARC 1200 mg/24 h	-	-	-	0	2–4	85 37 3	2 3 4
	CI ARC 2400 mg/24 h	-	-	-	22 0	2 3–4	100 85	2–3 4
Barrasa et al., 2020 [11]	CI CrCl = 40–130	7.9	-	-	-	-	-	-
	II CrCl = 60–130	-	18.5	3.3	85 1	2 4	86 49	2 4
	CI CrCl ˃ 130	2.8	-	-	-	-	-	-
	II CrCl ˃ 130	-	11.9	0.3	0	2–4	0	2–4
Soraluce et al., 2020 [14]	CI + noCRRT	3.35	-	-	-	-	86 50	2 4
	II + CRRT	-	-	-	52 0	2 4	-	-
	II + noCRRT	-	-	-	65 6	2 4	-	-
Taubert et al., 2017 [27]	CI	-	-	-	-	-	100	2–4
	II 1200 mg q 12 h	-	-	-	75	2–4	69	2–4
	II 300 mg q 6 h	-	-	-	-	-	87.5	2–4
De Pascale et al., 2015 [13]	CI	6.2	-	-	36.3	2	100	2–4
	II	-	10 Serum	1.7	18.2	45.1	82 33	2 4
			8.3 ELF					
De Pascale et al., 2013 [18]	CI	6	-	-	57	-	100	2–4
	II	-	9.4	1.4	14.28	2	76.1 44	2 4
Adembri et al., 2008 [20]	CI	-	11.5	-	87.5	2	100	1–2
	II	-	13.1	-	62.5	2	94.3	1–2
Barrasa et al., 2017 [19]	CI	3.8	-	-	-	-	-	-
Boselli et al., 2012 [23]	CI	7.1 Serum/ ELF	-	-	-	-	-	

ARC—augmented renal clearance, AUC—area under the curve for 0–24 h, T—time, CI—continuous intravenous infusion, ELF—epithelial lining fluid II—intermittent intravenous infusion, CrCl—creatinine clearance (mL/min/1.73 m^2^), C—concentration, Cmax—maximal concentration, Cmin—minimal concentration, Css—steady state concentration, h—hour, MIC—minimal inhibitory concentration in mg/L.

**Table 3:** Studies results in terms of clinical aspects, adverse events, and drug concentrations at effective and toxic concentrations.

Reference	Type of Infusion	% of pts with Cmin 10 mg/L	Side Effects	% of pts with C Constantly = 1–4 mg/L	Clinical Aspects/Observation
Santimaleeworagun et al., 2021 [24]	CI	99.6	ND	ND	ND
	II	14.3	ND	ND	ND
Taubert et al., 2017 [27]	CI	16	ND	30	ND
	II	4	ND	0	ND
Protti et al., 2016 [28]	CI	ND	Severe refractory lactic acidosis without hypoxia (high venous oxygen saturation)	ND	Microbiologic negativation after 3 days
Alvarez-Lerma et al., 2016 [22]	CI	ND	0	ND	Pulmonary samples negativation and invasive mechanical ventilation weaning
	II	ND	0	ND	Blood cultures became negative on the fifth day of treatment, but the patient showed a protracted respiratory clinical course with worsening of radiographic images
De Pascale et al., 2015 [13]	CI	ND	0	ND	81.8% (day 4 clinical improvement) 9% ICU mortality Alveolar diffusion = 98.8%
	II	ND	0	ND	72.7% (day 4 clinical improvement) 36.4% ICU mortality Alveolar diffusion = 87.1%
De Pascale et al., 2013 [18]	CI/II	ND	ND	ND	Alveolar diffusion = 100%
Boselli et al., 2012 [23]	CI	ND	0	ND	Alveolar diffusion = 97%
Tascini et al., 2011 [21]	CI	ND	0	ND	ND
	II	ND	12.5% (thrombcytopenia)	ND	ND

C—concentration, Cmin—minimal concentration, CI—continuous intravenous infusion, II—intermittent intravenous infusion, ND—not determined, ICU—intensive care unit, pts—patients, C = 1–4 mg/L is considered the effective concentration, Cmin ≥ 9–10 mg/L is considered the toxic concentration.

## Data Availability

Not applicable.
